# Risk factors for postpartum intrauterine device expulsion among women delivering at a tertiary Hospital in Uganda: a prospective cohort study

**DOI:** 10.1186/s40834-021-00153-w

**Published:** 2021-03-02

**Authors:** Joy Muhumuza, Richard Migisha, Joseph Ngonzi, Musa Kayondo, Godfrey Mugyenyi

**Affiliations:** 1 Department of Obstetrics and Gynecology, Mbarara University of Science and Technology, Mbarara, Uganda; 2 Department of Obstetrics and Gynecology, Kampala International University, Kampala, Uganda; 3 Department of Physiology, Mbarara University of Science and Technology, P.O Box 1410, Mbarara, Uganda

**Keywords:** Copper intrauterine device, Intrauterine device, Intrauterine device expulsion, Postpartum contraception, Postpartum intrauterine device, Postplacental intrauterine device, TCu380A intrauterine device

## Abstract

**Background:**

Postpartum intrauterine device (PPIUD) use refers to intrauterine device (IUD) insertion after delivery but within 48 h of birth. In Uganda, the general use of modern methods of contraception is low with < 1% of the women adopting the IUD as a method of contraception. An important limiting factor to increased uptake of immediate postpartum IUD insertion may be its expulsion rates which vary widely. There is minimal documentation PPIUD expulsion rates and factors associated with PPIUD expulsion during puerperium in Uganda.

**Objective:**

We aimed to determine the proportion of TCu380A (copper) intrauterine devices expelled by 6 weeks postpartum, and identify risk factors for expulsion among women delivering at Mbarara Regional Referral Hospital (MRRH) in southwestern Uganda.

**Methods:**

We conducted a prospective cohort study from September 1, 2014 to January 31, 2015 at MRRH. We administered a structured questionnaire to all participants, to capture data on their baseline demographic, clinical and obstetric characteristics. We followed up women who accepted the PPIUD insertions at 6 weeks post insertion for any IUD expulsion. We fit multivariable log binomial regression models to identify risk factors for IUD expulsion.

**Results:**

We enrolled 167 women who had PPIUDs inserted. Of the144 women who returned at 6 weeks for follow up, 13 (9%; 95%CI:4.9–15%) of them had the IUDs expelled. In the multivariable model, the significant risk factors for PPIUD expulsion were: IUD insertion more than 10 min post-delivery (aRR 8.1, 95%CI 1.26–51.98, *p* = 0.027) and bloody lochia flow of ≥15 days (aRR 8.5, 95%CI 1.47–48.47, *p* = 0.017).

**Conclusion:**

The cumulative expulsion rate of postpartum IUDs among women delivering at MRRH was low and comparable to expulsion rates in interval insertions. Longer duration from delivery to IUD insertions and longer duration of bloody lochia flow were key risk factors for postpartum IUD expulsion. More emphasis should be put on prenatal counseling for postpartum family planning to allow for postplacental IUD insertions, which are associated with lower expulsion rates.

## Introduction

Globally, approximately 220 million women in need of modern family planning methods are unable to access them for various reasons [1, 2]. The unmet need for family planning in sub-Saharan Africa, including Uganda is still very high (25 and 34% respectively) [3–5]. Across sub-Saharan Africa and Uganda, the IUD remains underutilized with just 1 and 0.5% of married women using it, respectively [5, 6]. The intrauterine device (IUD) is a highly effective, safe, reversible and long-acting type of contraception [7]. The CuT380A device is the most effective of IUDs with failure rate of 0.6–0.8 pregnancies per 100 women in the first 12 months of use and has the lowest cumulative pregnancy rate (2.2 per 100 women after 8 years) [8]. Postpartum intra uterine device (PPIUD) insertion bridges the limitations to the unmet family planning need by promoting IUD use. Offering postpartum insertions is one of the reasons for high uptake of the IUD in countries like China, Egypt, Mexico and Turkey where the IUD is the most popular reversible method of contraception [9]. The unique advantage of postpartum intrauterine contraception is that contraception is initiated early with a very low failure rate. Insertion of an IUD immediately after delivery has been recommended by WHO, as one of the safest and most effective method of reversible contraception for postpartum women who wish to either space or limit births, whether they are breastfeeding or not [8].

In Uganda, 25% of total births are less than 2 years apart [5], yet birth spacing is an important maternal and child health intervention [10]. For women with limited access to medical care, the time of delivery offers a unique opportunity to address their need for contraception if the delivery takes place in a healthcare facility. In addition, PPIUD may help avoid the discomfort related to interval insertion since insertion complaints caused by bloody lochia and cramping are masked [11]. Furthermore, effective utilization of family planning could avert approximately 32% of maternal deaths worldwide, by avoiding unintended premature pregnancies and abortions [12]. As previously reported [13], optimal utilization of modern contraceptive methods could result in approximately 85% decline in induced abortions in Uganda. In the immediate post-partum period, women are highly motivated to take up an effective contraception, as opposed to waiting for 6 weeks to initiate effective contraception. On the contrary, if the women are left to wait, they may conceive unintentionally or may not even return for contraception [14]. Appropriate times for IUD insertion in the immediate postpartum period include the postplacental (within 10 min after expulsion of the placenta), the immediate postpartum (within 48 h of delivery) and trans-cesarean IUD insertion (during a cesarean delivery), before the uterine incision is sutured.

An important limiting factor to increased uptake of immediate PPIUD insertion may perhaps be due to its expulsion rates, that range from as low as 5% to as high as 70 [15, 16]. Indeed, there has been skepticism in the recent past about PPIUD among some healthcare professionals with regard to the variable complication and expulsion rates cited in literature [1, 17]. Previous studies have found several factors to be associated with PPIUD expulsion, including: age [18], parity [19], mode of delivery [20] and timing of insertion [21]. There is minimal documentation on expulsion rates and risk factors of PPIUD expulsion in Uganda. In this prospective cohort study, we sought to determine the expulsion rate of PPIUD at 6 weeks postpartum and risk factors for PPIUD expulsion, among women delivering at Mbarara Regional Referral Hospital (MRRH), southwestern Uganda.

## Methods

### Study site and setting

We conducted a prospective cohort study from September 1, 2014 to January 31, 2015 at the maternity ward (labor, postnatal and operating theatre sections) of Mbarara Regional Referral Hospital (MRRH) in southwestern Uganda. The hospital serves as a referral centre for other ten district hospitals within the south-western region. The maternity ward of MRRH records approximately 10,223 total deliveries annually, with an average of 20–25 vaginal deliveries daily and a caesarean section rate of 30%. The hospital also has a family planning clinic which offers contraceptive services to an estimated 993 women annually and about 3% of these women take up interval IUD [22].

### Study population

Our study participants were women who delivered at MRRH (both vaginal and cesarean deliveries) during the 5 months’ study period, that fulfilled the inclusion criteria. We included women who met the WHO medical eligibility for PPIUD insertion [8], resided in Mbarara municipality (within 30 km radius), consented to participate in the study, and were willing to return at 6 weeks post IUD insertion for follow up. We excluded women with any of the following medical conditions: altered level of consciousness, post-partum hemorrhage (PPH), temperature > 38 °C during or after labor, pre-labor rupture of membranes > 18 h, obstructed labor, and uterine fibroids or malformations.

### Study procedures and sampling

We employed consecutive sampling technique to recruit eligible participants. We assessed eligibility by checking medical records of all women in early labor and those who had delivered vaginally a night before, to ascertain eligible participants without contraindications to PPIUD insertion, as well as checking the place of residence. We introduced the study to eligible participants through family planning health talks held daily on the maternity ward as part of routine practice.

Each eligible woman received individual counseling session about postpartum contraception where IUD was highlighted together with other options that included: mini-pills, bilateral tubal ligation, implants, lactation amenorrhea method and condoms. This enabled the women to make a voluntary and informed choice. The merits of each method, their common side effects and possible complications were explained comprehensively. Eligible participants were then approached individually to obtain an informed consent. Upon consent, the IUD was inserted within 48 h of delivery. Those who opted for an alternative method were referred to the family planning clinic at MRRH to receive the methods of their choice.

We used the following study instruments: a screening tool, a structured questionnaire and a PPIUD follow up card and form. We used the screening tool to counter check the eligibility of the women and to exclude those with contraindications to PPIUD insertion. We administered a structured questionnaire to capture data on socio-demographic, clinical and obstetric characteristics of the participants, including their data on family planning and gynecological history. The study participants were given health education on how best to recognize expulsions for example through the string length or even vivid expulsion, postpartum warning signs like bright red bleeding for which the woman needed to change her pad more than six times a day, unusual abdominal or pelvic pain (not after-birth pain) and unusual vaginal discharge or pain. The PPIUD follow-up card contained information on the date of insertion and follow up visit, date of removal of the IUD, the telephone number of the principal investigator and one of the research assistants. We used the PPIUD follow-up form to assess the postnatal experiences of the participants and it was filled at the follow-up visit. The follow up form was filled by asking questions and performing clinical examination. A speculum examination was then performed to check if the strings were visible or not and any discharge noted. The visible IUD strings were trimmed at approximately 3-5 cm. Participants whose strings were invisible at the cervical opening (os) and had not reported vivid IUD expulsion were sent for a uterine ultra sound scan to confirm expulsion. We assessed the study participants in whom the PPIUDs were inserted before discharge for comfort and followed them up at 6 weeks for expulsion. We emphasized the need for review at 6 weeks post-insertion. Participants were advised to call or comeback (any time before the set follow-up date) in case of any concern about the method. At 6 weeks, speculum or digital vaginal examination and/or abdominal/pelvic ultra sound scan were used to confirm IUD presence or expulsion. In addition, string shortening was done at the follow up visit.

### IUD insertion technique

All necessary instruments (CuT380A, sterile ring forceps, Sims speculum, povidone- iodine, kidney dish, head lamp and gauze pieces) were prepared. Infection prevention protocols were observed at all times by use of sterile equipment and the insertion was performed by a trained healthcare professional (midwife or medical doctor) using ring forceps. The IUD was inserted to the uterine fundus as confirmed by palpation with a hand placed on the abdomen overlying the fundus. The cervical os was then gently inspected with the Sims speculum for strings before the participant left the procedure room. To ensure data quality control, all the healthcare professionals involved in IUD insertion had undertaken a training program on PPIUD and interval IUD insertion by Population Services International (PSI) and Marie stopes International (MSI) and were certified competent. In addition, every 10th PPIUD insertion was supervised by a consultant Obstetrician/gynecologist.

### Study variables

Our primary outcome variable was IUD expulsion by 6 weeks post insertion. The independent variables were: socio-demographic factors (age, marital status, tribe, religion, level of education and occupation), obstetric and gynecologic factors (parity, birth weight of baby, duration of labor, mode of delivery, time lag from delivery to IUD insertion, menstrual flow and history of IUD expulsion), and postnatal factors (severe cramps in puerperium, resumption of sexual intercourse, breastfeeding, excessive vaginal bleeding in puerperium, use of herbs, douching, tummy tying, return of menses and heavy physical activity).

### Sample size and data analysis

We computed the sample size using the Fleiss formula for unmatched cohort studies, with the following assumptions: power of 80%, percentage of exposed with outcome of 25.9% and percentage of unexposed with outcome of 14.3%, based on the Mexican prospective cohort study where 25.9% of women who were multiparous expelled the PPIUD and 14.3% of primeparas expelled the PPIUD [19]. This gave a total sample size of 170 participants, after inflation for 10% loss to follow-up.

We entered data in EpiData (EpiData, Odende, Denmark) and later exported to Stata (StataCorp, College Station, Texas, USA) for analysis. We described baseline socio-demographic, obstetric, gynecological and clinical characteristics of participants. We determined the expulsion rate as the cumulative incidence by dividing the number of women who had the IUDs expelled by total number of women who came for follow up and expressed as a percentage. To determine the risk factors for expulsion of IUD, we performed univariable and multivariable analysis, using log binomial regression analysis. Our measure of association was risk ratios (RRs). All factors with *p*-value < 0.2 in univariable models were included in the multivariable models through backward stepwise method to determine the adjusted risk factors for PPIUD expulsion. Corresponding adjusted risk ratios (aRRs) and their 95% confidence intervals were reported. Risk factors were considered significant at a *p*-value of < 0.05.

## Results

**Fig. 1:**
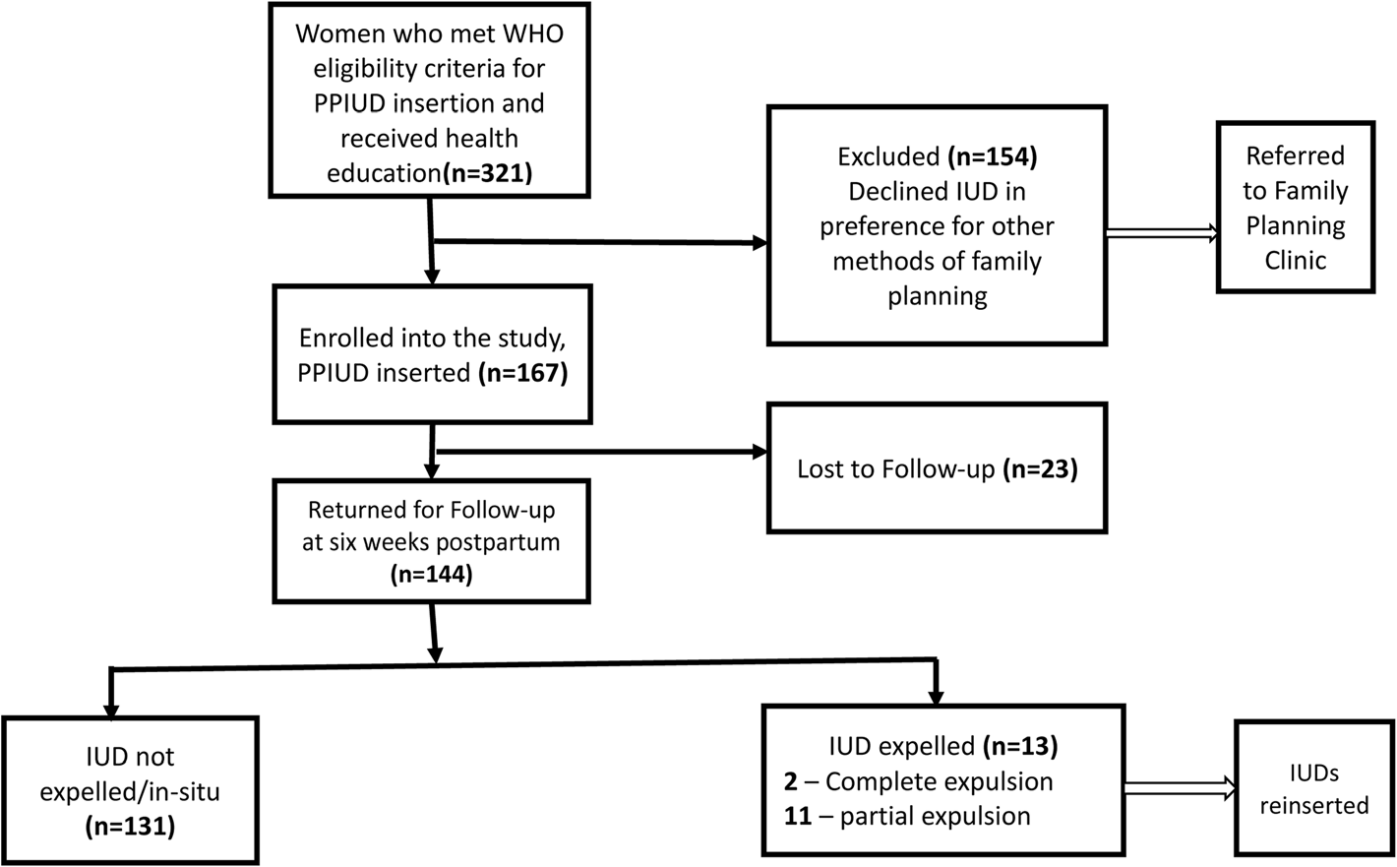
Flow diagram showing recruitment, enrollment and Intrauterine device (IUD) status at follow up of study participants

Of 324 women who received health education, we present results for 167 women who were enrolled into the study. The various reasons for non-participation into the study are depicted in Fig. 1.

### Baseline characteristics of study participants

Of the 167 study participants, most were aged 25–34 years (53.9%), married (91%), housewives (50.9%), had some formal education (94.6%) and were Christian (89.7%) (Table 1).

**Table 1:** Baseline Socio-demographic and obstetric Characteristics of study participants (*N* = 167)

Characteristics	Frequency (n)	Percentage (%)
**Age**		
18–24	44	26.3
25–34	90	53.9
> =35	33	19.8
**Education**		
None	9	5.4
Primary	64	38.3
Secondary	55	32.9
Tertiary	39	23.4
**Marital Status**		
Single /Divorced	15	9
Married	91	91
**Parity**		
One	26	15.6
Two	30	18
Three	34	20.4
Four	18.5	18.5
Five & above	46	27.5
**Mode of delivery**		
Vaginal	125	74.9
Caesarean section	42	25.1
**Baby condition at birth**		
Alive	166	99.3
Stillbirth	1	0.7
Birth weight (Bwt*) in kilograms		
< 2.5	12	7.2
2.5–3.9	96	57.5
> =4.0	59	35.3
**Time from delivery to IUD insertion (in minutes)**		
< =10	75	45.2
15–59	28	16.9
> =60	63	37.9
Median	20 (IQR,10–285)	
**Menstrual flow**		
Heavy	7	4.2
Normal	157	94
Scanty	3	1.8
**Associated cramps**		
Yes	60	35.9
No	107	64.1
**Ever used Intrauterine Device**		
Yes	11	6.6
No	156	93.4

*IQR* Inter-quartile range, *IUD* Intra-uterine device*;* Bwt*: Mean, 3.3 kg (SD ± 0.6).

The majority of the participants were multiparas (84.4%), had delivered vaginally (74.9%) and had live babies (99.3%) with a mean birth weight of 3.3 kg (SD ± 0.6). Seventy-five participants (45.2%) had IUD inserted within 10 min of delivery. Only eleven participants (6.6%) had ever used IUD for family planning and the mean duration of use was 4.0(SD ± 1.9) years.

Postnatal characteristics of the 144 participants who returned for follow-up are presented in Table 2. About half (51.4%) of the study participants had resumed sexual activities by the time of review; 9% of the participants had returned their menses by 6 weeks’ post- delivery. Few (12.5%) participants had resumed heavy physical activity by 6 weeks’ post-partum. History of herbal medicine use was reported in 15.3% of the study participants.

**Table 2:** Postnatal characteristics of study participants (*N* = 144)

Characteristic	Frequency	Percentage
**Resumption of sexual activities**		
Yes	74	51.4
No	70	48.6
**Return of menses**		
Yes	13	9
No	131	91
**Tummy tying**		
Yes	70	48.6
No	74	51.4
**Vaginal douching**		
Yes	18	12.5
No	126	87.5
**Use of herbs**		
Yes	22	15.3
No	122	84.7
**Resumption of heavy physical activity**		
Yes	18	12.5
No	126	88.5

### Expulsion rate of PPIUD by 6 weeks post-insertion

Among the 144 participants who had PPIUDs inserted, 13 had them expelled by 6 weeks post-insertion, for an expulsion rate of 9% (95%CI:4.9–15%), as seen in Fig. 1. Of the 13 participants who had PPIUD expelled, two (15.4%) had complete expulsion of the IUD within 2 weeks of insertion, while the remaining 11 (84.6%) had their IUDs partially expelled (with IUD stem visualized at the external cervical os on speculum exam) at 6 weeks (Fig. 1).

### Risk factors for PPIUD expulsion

The results for univariable and multivariable analysis for the risk factors for PPIUD expulsion are presented in Table 3. In the multivariable model, the significant risk factors for PPIUD expulsion were: IUD insertion between 11 and 59 min after delivery (aRR 8.11, 95%CI: 1.26–51.98, *p* = 0.027) and duration of bloody lochia flow> 15 days (aRR 8.45, 95%CI: 1.47–48.47, *p* = 0.017).

**Table 3:** Risk factors for PPIUD expulsion

	Unadjusted analysis	Adjusted analysis	
Variable	RR (95% CI)	Adjusted RR (95%CI)	***p***-value
**Age in years**			
18–24	1		
25–34	1.18 (0.22–6.16)		
≥ 35	2.79 (0.49–15.62)		
**Education Level**			
None	1		
Primary	0.88 (0.091–8.646)		
Secondary	1.00 (0.102–9.75)		
Tertiary	0.44 (0.035–5.522)		
**Birth weight (in kilograms)**			
< 2.5	1		
2.5–3.9	0.36 (0.35–3.71)		
≥ 4	1.24 (0.13–11.52)		
**Parity**			
Parity 1	1		
Parity 2	2.71 (0.26–28.37)		
Parity3	0.65 (0.04–11.11)		
Parity4	0.70 (0.04–11.96)		
5 & above	4.03 (0.46–35.29)		
**Time from delivery to IUD insertion (in minutes)**			
≤ 10 min	1	1	
11-59 min	8.95 (1,61–49.81)	**8.11 (1.26–51.98)**	**0.027**
≥ 60 min	4.86 (0.94–25.19)	4.78 (0.86–26.43)	0.070
**Duration of menses in days**			
≤ 3			
4	1		
> =5	1.23 (0.34–4.49)		
**Past IUD use**	1.61 (0.35–7.30)		
No	1		
Yes	1.1 (0.13–9.53)		
**Resumption of sex**			
No	1		
Yes	3.60 (0.95–13.69)		
**Duration of bloody lochia flow in days**			
0–7	1	1	
8 to 14	1.91 (0.48–7.53)	1.35 (0.33–5.53)	0.924
≥ 15	7.37 (1.45–37.45)	**8.45 (1.47–48.47)**	**0.017**

## Discussion

The expulsion rate of 9% observed in our prospective study is consistent with findings from several studies that have reported expulsion rates that range from 5 to 15% [23–27]. A lower expulsion rate of 3.8% than ours was reported in India, probably because the study in India relied on self-report of IUD expulsion, and therefore subjectively determined. In our study we used both clinical exam and sonographic criteria to ascertain IUD expulsion. Higher expulsion rates of 17 and 16% have been reported in USA [28] and Mexico [19] respectively. The possible reason for the higher expulsion rates in these studies than ours, could be because of the longer follow-up time of 3 to 6 months. In our study, we followed up women for only 6 weeks postpartum. Similarly, higher expulsion rates than ours have been observed in studies that used levonorgestrel IUD [17, 29]. The higher expulsion rates of levonorgestrel IUD may possibly be attributed to the use of levonorgestrel-releasing IUD inserter for postplacental insertions, as opposed to our study in which we used ring forceps for IUD insertions, and therefore had higher chances for high fundal placement [30]. To decrease IUD expulsion rates, high fundal placement is recommended [30, 31].

The expulsion rate in the current study is comparable to that observed in interval IUD insertions. This has important implications for family planning uptake and reproductive health in Uganda. Given that Uganda has a high unmet need for family planning [5, 32], there is need to emphasize prenatal counseling for postpartum family planning as this will facilitate postplacental IUD uptake. In addition, the Ministry of Health should consider conducting in-service training of all health personnel who conduct deliveries, in postplacental IUD insertions, so as to ensure 24-h availability of the service providers with skills to insert postplacental IUDs.

In this study, the time lag from delivery to IUD insertion and duration of lochia flow were significantly associated with postpartum IUD expulsion, in agreement with previous findings [15, 21, 28]. We found that women who had IUDs inserted 11–59 min after delivery had a higher risk than those who had the IUDs inserted within 10 min post-delivery. This could be explained by the fact that within 10 min of delivery, the cervix is still fully open, making placement of the IUD high in the uterine fundus much easier compared to insertion at a later time [15]. This finding further underscores the need for routine prenatal counselling of women on post-partum post placental IUD insertions to reduce on expulsion rates associated with late insertions.

Women who experienced bloody lochia flow for 15 or more days had higher risk for IUD expulsion compared to their counterparts who experienced fewer days of bloody lochia flow. This is probably due to the fact that bloody lochia flow is associated with increased uterine contractions, which may be induced by the presence of a foreign body in the uterus [33], and this may lead to IUD expulsion. Although this association is a new finding, these data call for ensuring that women who experience prolonged bloody lochia flow following postpartum IUD insertion, be encouraged to return early for follow up (within 6 weeks), in order to detect any spontaneous expulsion.

## Limitations

Our study has some limitations worth mentioning. First, the main limitation for our study is loss to follow-up. Twenty-three (14%) of the 167 participants were lost to follow-up. Nevertheless, the retention rate was good enough (86%) not to affect the power of the study. In addition, there was no statistically significant difference in the baseline characteristics of participants who were lost to follow-up and those who were retained in the study. Second, our follow-up period of 6 weeks was relatively shorter compared to what is reported in background literature [9]. We recommend further longitudinal studies, with longer follow-up periods for PPIUD expulsions in our setting in Uganda. Finally, we used only CuT380A IUDs in our study because they are the ones recommended for use for PPIUD insertions, by the Ministry of Health in Uganda. Therefore, our findings may not be generalizable beyond the population of women using CuT380A IUDs, such as those using Levonorgestrel IUDs. The major strengths of our study lie in its prospective nature and the fact that we objectively assessed expulsion of PPIUDs using both clinical examinations and ultrasound scan when clinically indicated.

## Conclusion

In conclusion, the cumulative expulsion rate of postpartum IUD among women delivering at MRRH was low and comparable to expulsion rates in interval IUD insertions. Delayed time from delivery to IUD insertion and prolonged duration of bloody lochia flow were the key risk factors for postpartum IUD expulsion during puerperium, among women delivering at MRRH in southwestern Uganda. Our findings support routine prenatal counseling for postpartum family planning to allow for post-placental IUD insertions, which are associated with lower expulsion rates.

## Data Availability

The datasets generated and analyzed during the study are available from the 1st author upon reasonable request.

## Abbreviations

aRR: Adjusted risk ratio
CI: Confidence interval
FRC: Faculty Research Committee
IQR: Interquartile range
IUD: Intrauterine device
MRRH: Mbarara Regional Referral Hospital
MSI: Marie stopes International
MUST: Mbarara University of Science and Technology
PPH: Postpartum hemorrhage
PPIUD: Postpartum intrauterine device
PSI: Population Services International
REC: Research Ethics Committee
RR: Risk ratio
SD: Standard deviation
WHO: Word Health Organization
